# Ecological Dynamics and Functional Classification of *Nanosynbacter lyticus* Strain TM7x in the Human Oral Microbiome: A Literature Review

**DOI:** 10.3390/microorganisms14071447

**Published:** 2026-06-30

**Authors:** María de Lourdes Rodriguez Coyago, Isabel Narcisa Berrezueta Reyes, Marco Miguel Vega García, Esteban Fernando Lima Tola, Wilson Daniel Bravo Torres, Jacinto José Alvarado Cordero

**Affiliations:** 1 Postgrado de Rehabilitación Oral, Facultad de Odontología, Universidad de Cuenca, Cuenca 010107, Ecuador; maria.rodriguezc84@ucuenca.edu.ec (M.d.L.R.C.); isabel.berrezueta99@ucuenca.edu.ec (I.N.B.R.); marco.vega@ucuenca.edu.ec (M.M.V.G.); wilson.bravo@ucuenca.edu.ec (W.D.B.T.); 2 Departamento de Microbiología y Diagnóstico, Facultad de Odontología, Universidad de Cuenca, Cuenca 010107, Ecuador; 3 Grupo de Investigación en Rehabilitación Oral (GIRO), Facultad de Odontología, Universidad de Cuenca, Cuenca 010107, Ecuador; 4 Grupo de Investigación en Periodoncia e Implantología Oral (GIPIO), Facultad de Odontología, Universidad de Cuenca, Cuenca 010107, Ecuador

**Keywords:** Saccharibacteria, *Nanosymbacter lyticus*, TM7x, oral microbiome, ecology

## Abstract

The TM7x strain is a genetic variant of the bacterium *Nanosynbacter lyticus*, which belongs to the Saccharibacteria phylum within the Candidate Phyla Radiation (CPR) or Patescibacteria group. Its biology differs significantly from that of other bacterial phyla, and its ecological role in the oral cavity remains largely undefined. Through a organyzed and comprehensive literature review, we aim to define the role this bacterium plays within the oral ecosystem. We identified relevant studies from primary sources, including scientific articles from preclinical and clinical studies obtained from three digital databases. The bacterial strain TM7x is an obligate epibiont that exhibits autonomous energy metabolism and utilizes a type IV pili system to adhere to its direct host, *Schaalia odontolytica*. It interacts with its host in two stages: initially as an epipatobiont and subsequently as an episymbiont. TM7x plays a complex ecological role by modulating the host’s metabolism and structure toward a less virulent phenotype resistant to phage attack, while also influencing the human host through immunomodulation and tissue protection. This organism has transitioned from being considered ‘biological dark matter’ to a key model for understanding coevolution within the human microbiome. Its ability to protect the host from phages, induce protective biofilms, and suppress destructive inflammatory responses suggests its potential role as a speculative modulator of human oral microbiome homeostasis, although direct clinical confirmation in human subjects is still lacking.

## 1. Introduction

*Nanosymbacter lyticus* TM7x is the first cultivated member of the phylum Saccharibacteria, part of the Candidate Phylum Radiation (CPR)—now recognized as the superphylum Patescibacteria—a vast and genetically diverse monophyletic group estimated to account for over 25% of all bacterial diversity [1,2]. These organisms are characterized by an epibiotic lifestyle, ultra-small cell sizes (200–500 nm), and highly reduced genomes lacking most essential biosynthetic pathways [2]. For TM7x, its biological existence is fundamentally defined by its dependence on a host from the genus *Actinomyces*, specifically *Schaalia odontolytica* strain XH001 [3]. Whether this relationship is inherently deleterious (epipatobiont) or provides compensatory benefits that preserve oral health (episymbiont) remains a central question in the field. This inquiry is underscored by evidence suggesting that Saccharibacteria (TM7) may contribute to periodontal pathogenesis [1,4,5,6]. Consequently, this structured review synthesizes primary evidence from the past decade to define the ecological role of *N. lyticus* TM7x within the oral ecosystem.

## 2. Methodology

### 2.1. Search Strategy and Selection Criteria

A systematic search was conducted to identify primary studies regarding the physiology, genomics, and ecological interactions of *Nanosynbacter lyticus* strain TM7x. The PubMed, Scopus, and Embase databases were queried for literature published between 2015 (marking the initial isolation of TM7x) and 2025.

Inclusion criteria encompassed: (i) studies utilizing the cultivated strain TM7x or its specific host (*S. odontolytica* XH001); (ii) molecular or transcriptomic analyses of the host-epibiont relationship; (iii) clinical studies correlating Saccharibacteria abundance with human oral health or disease parameters; and (iv) functional assays involving animal models or human cell cultures.

Exclusion criteria were applied to studies solely reporting 16S-rRNA diversity profiles without functional analysis, as well as articles focused exclusively on environmental Saccharibacteria unrelated to the human microbiome.

### 2.2. Review Workflow and Data Synthesis

The selection process followed a structured workflow:

- Identification: 351 records were initially identified through database searches.
- Screening: 165 unique entries remained after deduplication and title/abstract screening for thematic relevance.
- Eligibility: 44 full-text articles were rigorously assessed for functional or experimental data on TM7x.
- Inclusion: 28 key articles were selected for qualitative synthesis. Given the fundamental heterogeneity in the experimental designs of the primary literature—ranging from comparative genomics to animal models—a qualitative approach was robustly selected to conceptualize the biological and ecological mechanisms of strain TM7x, as the diverse data structures preclude a quantitative meta-analysis. Furthermore, it is critical to clarify that this study is designed as a structured narrative review based on a systematic search strategy rather than a formal systematic review framework. Consequently, no formal assessment of study quality or risk of bias was performed across the retrieved literature, which represents a recognized methodological limitation of this synthesis. Data extraction and visualization were performed using Microsoft Excel and PowerPoint (v. 2021).

### 2.3. Data Extraction

To ensure a systematic, unbiased, and transparent synthesis of the retrieved literature, data extraction was executed independently by the investigators utilizing a standardized matrix protocol. From the 28 primary studies definitively included in this review (Table 1), data were systematically categorized and extracted across three core conceptual domains:

1. Genomic and Metabolic Architecture: Extraction focused on the genomic constraints of *Nanosymbacter lyticus* strain TM7x (such as its ~700 kb ultra-reduced genome), specific metabolic auxotrophies—including the complete absence of de novo biosynthesis pathways for lipid precursors and all 20 essential amino acids—and the active bioenergetic mechanisms retained for horizontal transmission, specifically glycolysis and the Arginine Deiminase System (ADS).
2. Host-Epibiont Kinetic Dynamics: We compiled detailed qualitative and quantitative metrics mapping the four-phase life cycle of TM7x: Initial Encounter, Lytic/Death Phase, Recovery, and Stable Symbiosis. The dataset integrates host structural parameters (*Schaalia odontolytica* XH001 cell elongation, hyphae-like formation, and cell-wall thickening) with key epibiont traits, including Type IV pili regulation and budding replication dynamics.
3. Ecological and Immunomodulatory Dynamics of TM7x: Data extraction targeted community-level structural modifications, specifically Autoinducer-2 (AI-2) quorum-sensing dynamics mediated by *lsrB* and *luxS* loci, and the mechanisms underlying the “epibiotic shield” against viral predation—such as phage LC001 adsorption rates and receptor downregulation. At the human–host interface, we systematically extracted parameters of immune signaling attenuation, including Toll-Like Receptor 2 (TLR2) clustering, pro-inflammatory cytokine suppression, like tumor necrosis factor alpha (TNF-α), and in vivo markers of alveolar bone preservation in murine models.

Following consensus-based cross-checking and harmonization, the extracted data were structured into variables. These variables directly informed the qualitative thematic synthesis and the conceptual design of the dynamic models.

## 3. Results

### 3.1. Genomic Architecture and Metabolic Constraints of Strain TM7x

The ability of *Nanosymbacter lyticus* TM7x to persist within the oral microbiome, despite severe genetic deficiencies, underscores its extreme evolutionary specialization [1,2]. Its genome (~700 kb) is among the smallest recorded for an extracellular bacterium [2]. This genomic reduction has led to the loss of core biosynthetic pathways for amino acids, lipids, vitamins, and cell wall precursors [7].

TM7x is nearly entirely auxotrophic; genomic analysis indicates it cannot synthesize any of the 20 essential amino acids de novo, nor the vitamin cofactors required for most enzymatic functions [7,8]. In contrast to its host, *S. odontolytica* XH001—which possesses over 123 functional metabolic pathways—TM7x operates with a minimal repertoire of only 20 pathways and approximately 151 identified chemical reactions [3,9]. This metabolic disparity necessitates intimate physical contact; TM7x must “harvest” molecular building blocks directly from the host’s surface or cytoplasm via specialized transporters highly expressed during stable symbiosis [9].

**Figure 1:**
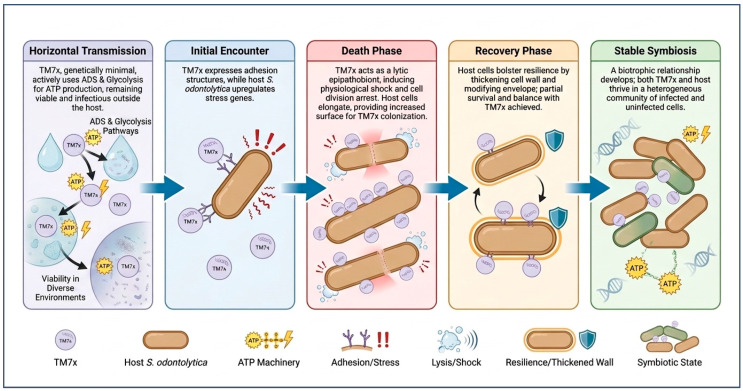
Ecological dynamics of *N. lyticus* strain TM7x in the oral cavity. Note: The scientific illustration depicting the “Life Cycle of TM7x” was generated using the FigureLabs Core Engine (2024), an AI-powered scientific illustration plataform, based on detailed textual descriptions provided by the authors.

Despite this minimalism, TM7x selectively retains energy-generating machinery. Recent evidence demonstrates that TM7x employs two ATP-generating pathways: the ADS and glycolysis [10]. These ensure viability and infectivity during horizontal transmission—a critical life-cycle phase when the epibiont dissociates from its host [10]. Notably, ammonia (NH_3_) production via ADS is not merely metabolic waste but an ecological strategy to neutralize local pH, thereby protecting both TM7x and its host in acidogenic microenvironments [11] (Figure 1). This retention of bioenergetic pathways suggests that TM7x is an active strategist capable of maintaining basal metabolic activity to ensure environmental persistence, a trait that contrasts with its traditional classification alongside other generic TM7 lineages frequently linked to active periodontal or mucosal destruction [4,5,6].

### 3.2. Dynamics of the Epibiotic Interaction: The TM7x Life Cycle

The relationship between *N. lyticus* TM7x and *S. odontolytica* XH001 is dynamic, progressing through stages that fundamentally alter the physiology of both partners. When TM7x is introduced to “naive” host populations, the interaction follows a predictable trajectory from acute virulence to stable coexistence [9].

This interaction is categorized into four phases: initial encounter, death phase, recovery, and stable symbiosis. Each stage is marked by distinct shifts in gene expression and morphology (Table 2). The initial encounter involves the expression of stress-response genes in the host (basibiont) and adhesion structures in the epibiont. During the death phase, TM7x acts as a lytic epipatobiont; the host undergoes physiological shock, disrupting cell division and resulting in elongated *Actinomyces* cells that provide an expanded surface area for colonization. While this resembles necrotrophic parasitism, the recovery phase highlights host resilience. By diverting energy into cell-wall thickening and envelope modification, a subpopulation survives to establish an equilibrium. In this stable symbiotic stage, the relationship becomes biotrophic, allowing both organisms to thrive within a heterogeneous community [9,10] (Figure 1).

### 3.3. Host Range and Genomic Specificity

Host-range mapping (2020) characterized the susceptibility of *Actinomyces* strains to TM7x at genomic and physiological levels, revealing high host specificity [13]. This suggests that susceptibility is governed by specific genetic determinants rather than being a universal trait, reflecting prolonged co-evolution.

Physiologically, two host types are recognized:

1. Permissive hosts (e.g., XH001): These exhibit a “growth-decline-recovery” response, undergoing severe initial stress and phenotypic changes before achieving symbiosis.
2. Non-permissive/Resistant hosts: These allow TM7x propagation without undergoing a lytic phase or significant morphological distortions (e.g., elongation), suggesting intrinsic resistance to TM7x-induced cytopathic effects [13].

Comparative genomics indicates that permissive hosts are enriched in genes for cell-envelope glycoprotein modification (e.g., glucosamine-PI de-N-acetylase), which facilitates TM7x attachment. Conversely, resistant hosts often carry variants in surface proteins and transport systems (e.g., secA, ugpC) that may preclude effective anchoring or mitigate infection-induced stress [12]. However, it is fundamental to underscore that the molecular coupling described between TM7x and *Schaalia odontolytica* XH001 represents a highly specific phenotype; other strains within the host genera exhibit drastic variations in susceptibility or complete non-permissiveness to epibiont colonization [12,13,29]. To date, the association of specific cell-envelope or genetic determinants with these resistance or permissiveness profiles remains purely correlative, relying predominantly on comparative sequencing of a restricted number of strains [13,29]. Due to the intrinsic difficulties in genetically manipulating these co-dependent systems, most candidate genetic determinants have yet to be functionally validated. Consequently, developing targeted genetic editing systems (e.g., CRISPR-Cas) to execute knockout or complementation assays of key target genes—such as *secA*, *ugpC* [13], and rhamnose biosynthesis pathways [9]—is an indispensable priority to rigorously confirm the mechanisms governing host range and the physical anchoring of *Nanosymbacter lyticus* [12,13].

### 3.4. Biofilm Modulation and AI-2 Quorum Sensing

TM7x interaction transcends the cellular scale, altering microbial community architecture. Notably, TM7x-XH001 co-cultures produce significantly denser biofilms with greater biovolume than XH001 monocultures. This modulation is mediated by the Autoinducer-2 (AI-2) quorum-sensing system [14]. In XH001, the most strongly induced gene upon association is *lsrB*, which encodes an AI-2 periplasmic binding protein [30]. While the *lsr* system in *Actinomyces* differs from the canonical Proteobacterial model, it acts as a master regulator of the response to TM7x. Deletion of *lsrB* or *luxS* does not prevent physical adherence but completely abrogates the symbiosis-induced increase in biofilm formation [14]. This structural reinforcement may serve as a defense mechanism against environmental stressors while simultaneously providing a stable niche for TM7x replication.

### 3.5. The Epibiotic Shield: Protection Against Bacteriophages

The strongest evidence defining TM7x as a conditional mutualist is its ability to shield the host from viral predation in phage-dense oral environments [15]. Using the lytic phage LC001 as a model, studies show that TM7x confers near-total phenotypic resistance to *S. odontolytica* XH001 [12]. This ‘epibiotic shield’ operates through a multifaceted mechanism: (i) Reduced Phage Adsorption: TM7x colonization plummets phage binding from over 90% to less than 20% [12]; and (ii) Receptor Downregulation: TM7x triggers the downregulation of genes synthesizing cell-wall polysaccharides (CWP) and teichoic acids—the primary attachment sites for LC001 [12,13]. This surface remodeling suggests a generalized defense mechanism against other phages sharing similar host receptors [13], preventing host extinction and enabling post-predation population recovery [12]. Furthermore, this interaction modulates broader community architecture via *luxS*- and *lsrB*-mediated Autoinducer-2 (AI-2) quorum sensing, inducing denser biofilms with increased biovolume [14]. The reinforced extracellular matrix acts as a three-dimensional physical barrier that restricts virion diffusion within the oral ecosystem [14,15]. Nevertheless, given the vast, uncharacterized viral diversity in the human oral cavity, future studies utilizing natural salivary viromes are required to confirm the universal scale of this protective symbiosis.

### 3.6. Immunomodulation and Human Host Interaction

Although observational studies associate TM7x with periodontitis [4,5,16], vaginosis [17], and inflammatory bowel disease [18], experimental data reveal a more nuanced, immunomodulatory role. In vitro, TM7x actively silences host-triggered inflammatory signals; while *Schaalia odontolytica* alone induces robust TNF-α production in human macrophages, co-infection with TM7x significantly suppresses this pro-inflammatory response [30]. Mechanistically, TM7x utilizes Type IV pili to induce TLR2 receptor clustering in gingival epithelial cells, dampening downstream signaling cascades and reducing cytokine production. Furthermore, TM7x can survive within epithelial lysosomes, suggesting a capacity for long-term immune evasion [19]. In vivo evidence from murine periodontitis models supports this stabilizing role, where TM7x attenuation of host pathogenicity downregulates genes essential for collagen degradation and sialic acid utilization, ultimately reducing alveolar bone loss [20].

Despite these insights, current evidence relies heavily on reductionist in vitro systems and a single ligature-induced murine model. These designs fundamentally fail to replicate the architectural complexity, salivary flow, and cellular diversity of the human periodontium, particularly given the well-documented discrepancies between murine and human periodontal immunology. Absent human prospective clinical trials, the therapeutic, anti-inflammatory capacity of TM7x remains speculative. These preclinical milestones establish strong conceptual foundations but urgently require validation through human observational cohorts.

### 3.7. Ecological Synthesis: Epipatobiont or Episymbiont?

The ecological balance of the TM7x interaction remains inherently dualistic. From an epipatobiont perspective, TM7x exhibits clear parasitic traits, including initial lethality toward naïve hosts [21], absolute metabolic auxotrophy [1,11,13], and a marked numerical expansion in diseased states [1,5]. Conversely, from an episymbiont perspective, cumulative evidence suggests a regulatory role; by conferring phage resistance [12], reinforcing biofilm matrix [14], and attenuating pathobiont virulence [20,30], TM7x effectively functions as a “microbiome tamer.” Consequently, while TM7x imposes a metabolic burden at the cellular level, it provides community-level benefits that may ultimately favor mammalian host homeostasis.

**Figure 2:**
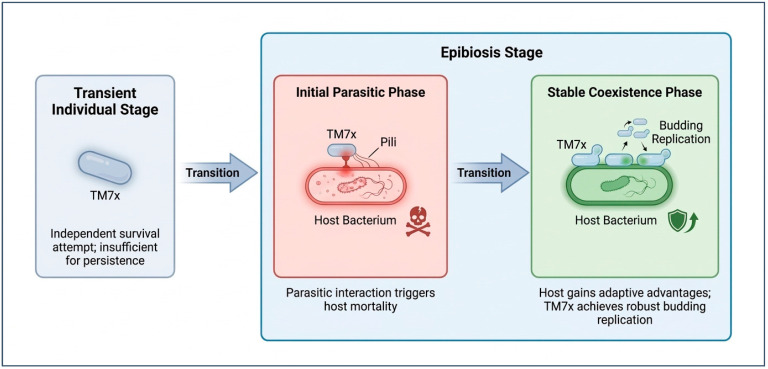
Ecological Lifecycle of *N. lyticus* TM7x in the Oral Microbiome. Note: The scientific illustration depicting the “Life Cycle of TM7x” was generated using the FigureLabs Core Engine (2024), an AI-powered scientific illustration plataform, based on detailed textual descriptions provided by the authors.

However, interpreting TM7x as a homeostasis-promoting agent currently relies on surrogate protective variables validated only in vitro and in murine models. The biomedical literature lacks longitudinal or interventional studies demonstrating that TM7x abundance translates into measurable improvements in human clinical parameters. Therefore, the proposed positive ecological balance outlined here (Figure 2) represents a preliminary mechanistic model, whose clinical validity remains contingent upon future prospective epidemiological studies and human clinical trials.

#### Metabolic Burden, Parasitic Costs, and Pathogenic Cross-Feeding

While the systemic outcome of the TM7x–*Schaalia odontolytica* interaction often aligns with homeostasis, a granular analysis reveals localized parasitic costs and ecological risks [1]. At the cellular level, TM7x colonization is highly exploitative. Complete auxotrophy for all 20 essential amino acids and lipid precursors turns the epibiont into an obligate metabolic sink [7,8]. To sustain TM7x, the host must undergo profound transcriptional reprogramming, diverting bioenergetic resources toward cell-wall thickening and the upregulation of membrane transporters [9,28]. This structural compensation imposes a chronic metabolic burden, drastically reducing the baseline growth rate of infected host strains compared to naïve counterparts [27].

Beyond individual host strains, TM7x introduces potential vulnerabilities within the oral polymicrobial architecture through three distinct mechanisms. First, the acute host lysis characterizing initial encounters releases intracellular necrotic debris into the local matrix, potentially providing assimilable nutrients for fastidious periodontal pathobionts, such as “red complex” species [27]. Second, while ammonia production via the ADS protects against caries by neutralizing organic acids, excessive nitrogenous byproducts in deep periodontal pockets can alkalinize the microenvironment, favoring the growth of tissue-destructive, proteolytic pathogens [10,11]. Finally, the denser biofilms induced via AI-2 quorum sensing may inadvertently act as physical sanctuaries, shielding co-aggregated pathogens from host immune clearance and antimicrobials [14,15]. Therefore, this ecological balance is strictly context-dependent, and under specific environmental pressures, TM7x-mediated cross-feeding and biofilm remodeling could facilitate, rather than restrict, dysbiosis [20,30].

### 3.8. Ecological Role of N. lyticus (TM7x) Across Oral Pathologies

Although clinical evidence associates oral TM7 expansion with mucosal infections, a causal relationship in periodontal destruction remains unestablished. While TM7 abundance correlates with periodontitis severity [6,21,22], declines post-therapy [23], and localizes near the crevicular epithelium [24], *Nanosymbacter lyticus* TM7x may actually act as a virulence biocontrol agent [1,14,20]. In vivo, the TM7x–*Schaalia odontolytica* complex significantly attenuates gingival inflammation and alveolar bone loss compared to colonization by the basibiont XH001 alone [20]. This reduced pathogenicity aligns with the downregulation of host bacterial virulence genes mediating adhesion to collagen and sialic acid residues [20]. Concurrently, transcriptomic profiling shows that *S. odontolytica* undergoes intense metabolic reprogramming during recovery from parasitism, upregulating stress-response genes and membrane transporters to counteract the epibiont [9]. Taken together, these data suggest that TM7x suppresses host virulence by forcing bioenergetic reallocation, thereby mitigating tissue damage and modulating host immunoactivation.

Beyond periodontitis, the physiological traits of TM7x point to regulatory roles in other oral ecosystems. In dental caries, TM7x expression of the ADS yields ammonia, neutralizing acidogenic microenvironments and potentially protecting tooth structures from demineralization [10,11]. In endodontic infections, where *Schaalia* strains frequently colonize necrotic root canals [3], TM7x-mediated AI-2 quorum sensing expands biofilm thickness [14] and suppresses macrophage activation [30], altering biofilm persistence and periapical inflammation. Finally, in peri-implantitis, the downregulation of collagen-binding proteins suggests that this epibiont may attenuate peri-implant alveolar bone loss, mirroring its protective dynamics observed in murine periodontitis models [20].

Despite these promising mechanistic insights, translating the protective role of TM7x into definitive clinical evidence faces severe methodological limitations. Current data rely heavily on reductionist, binary in vitro co-cultures and a single ligature-induced murine model, which fail to replicate the complex polymicrobial competition, salivary flow, and cellular diversity of the human periodontium. Furthermore, a pronounced model-organism bias persists, as these findings represent strain-specific features of TM7x rather than universal ecological laws across the highly diverse Saccharibacteria phylum. To conclusively demonstrate a preventive role in oral pathogenesis, future research must shift from rodent models toward multi-species biofilms integrating “red complex” pathobionts under dynamic microfluidic flow conditions. Most importantly, it is a clinical priority to establish human longitudinal cohorts and prospective clinical trials to determine whether early childhood colonization by the TM7x–Schaalia consortium naturally correlates with long-term resistance to dental caries and periodontitis.

## 4. Discussion

Saccharibacteria (formerly candidate division TM7) are ubiquitous obligate epibionts whose genomes have been identified across diverse environmental and mammalian niches [1]. As common constituents of the human oral, vaginal, cutaneous, and intestinal microbiomes [1,4,29,30], their abundance often increases significantly during mucosal inflammatory diseases [16,17,24]. Consequently, this group has traditionally been classified as putative pathogens. However, due to the historical recalcitrance of TM7 to in vitro cultivation, causal research is essential to elucidate its precise role in inflammatory pathogenesis.

*Nanosymbacter lyticus* strain TM7x, the first cultured representative of the phylum, reproduces via budding [1,30]. During horizontal transmission, daughter cells can dissociate from their host, *S. odontolytica*, to colonize new bacterial partners [27]. Recent findings by Nahar et al. (2026) [10] suggest that this phase of metabolic autonomy is supported by the combined expression of the ADS and glycolytic pathways for ATP generation. Nevertheless, these processes are insufficient for long-term viability, necessitating a stable association with an Actinobacterial host [14,24]. During this epiparasitic interaction, TM7x establishes a highly dynamic relationship with its host (XH001), modulated by physical attachment and environmental factors such as oxygen and nutrient availability [28]. Both partners undergo significant morphological and physiological shifts, including host cell elongation and cell-wall thickening under nutrient-rich conditions [24]. These changes are accompanied by the differential expression of over 300 genes, with those related to transport and stress responses exhibiting the highest upregulation [9,28]. As described by Bor et al. (2018) [27], the infection of a naïve host is initially characterized by an acute lytic phase—driven by an overwhelming epibiont load (exceeding 50 cells per host)—followed by the rapid development of reduced host susceptibility and the establishment of long-term stable symbiosis (Figure 1). This transition is hypothesized to result from rapid host evolution; genomic sequencing of stable lineages has revealed multiple mutations in transporter and regulatory genes that potentially confer partial protection against lethal infection [27].

The dual nature of *N. lyticus* TM7x reflects an ecological adaptation common among obligate symbionts with reduced genomes. In the volatile oral environment, where bacterial hosts face pressure from viral predation, antibiotics, and the immune system, association with an epibiont may constitute a vital survival strategy. This relationship can be interpreted through the lens of kin selection or community-level benefit: while individual cells may perish during initial infection [27], the resulting population achieves greater resilience and stability within the biofilm [14]. Furthermore, Dong et al. (2024) [31] demonstrated that TM7x infection induces the formation of host lipid droplets under environmental stress, potentially enabling *S. odontolytica* to withstand oxidative challenges [24,31,32]. Additionally, the capacity of TM7x to modulate human immune signaling suggests that Saccharibacteria have evolved not only to exploit their bacterial hosts but also to protect the shared ecological niche from excessive inflammatory destruction [14,20,30].

A critical, unresolved dilemma in Saccharibacteria ecology is whether their numerical expansion during disease—reaching up to 21% of the community [1,30]—constitutes an etiological driver of tissue destruction or merely an opportunistic bloom facilitated by the microenvironmental conditions of dysbiosis (such as increased Actinomyces biomass and an abundance of inflammation-derived nutrients) [1,27]. Addressing this question requires distinguishing cell-level effects from community-level effects [20,27]. While TM7x exerts an initial, metabolically costly and even lethal parasitism on individual cells of *S. odontolytica* [9,27], at the polymicrobial ecosystem level, it acts as an allosteric modulator, dampening the overall virulence of the bacterial consortium and halting alveolar bone loss [20]. Therefore, its increase in periodontitis should not be interpreted *a priori* as direct pathogenic causality, but potentially as an ecological feedback mechanism attempting to mitigate hyperinflammation [20,30]. Additionally, emerging evidence challenges the preconception that a high abundance of TM7 predicts periodontal disease. Indeed, a recent study comparing type 2 diabetic subjects with and without periodontitis found that TM7 abundance was significantly lower in the diabetic group with periodontitis, while remaining elevated in healthy and gingivitis-affected subjects. These findings contest the role of TM7 as a universal predictor of periodontitis. However, the study’s limitations—including a small sample size, lack of longitudinal glucose monitoring, and the measurement of total TM7 rather than specific strains like TM7x—must be considered. Given the high genotypic variability within the TM7 lineage [29], ecological responses are likely strain specific.

Collectively, preclinical and clinical data suggest that Saccharibacteria may not be direct drivers of inflammation [19,20,33]. Instead, their expansion may represent an ecological response to host (Actinobacteria) abundance or a systemic attempt to mitigate the virulence of “red complex” pathobionts through indirect interactions. This perspective shifts the clinical interpretation of these organisms, suggesting their potential future use as probiotics or replacement therapy agents to restore homeostasis in chronic periodontal disease.

In summary, the oral ecology of *N. lyticus* TM7x involves two distinct stages: a transient individual stage of independent survival—insufficient for persistence—followed by the defining stage of epibiosis. This latter stage encompasses an initial parasitic phase that triggers host mortality, transitioning into a stable coexistence where the host gains adaptive advantages while the epibiont achieves robust replication via budding (Figure 2).

## 5. Biases and Limitations

Current functional evidence in Saccharibacteria research is constrained by significant methodological, epidemiological, and ecological boundaries. First, a profound “model-organism bias” exists due to the historical monopolization of functional assays by *Nanosymbacter lyticus* strain TM7x. Given the vast phylogenetic diversity within the Candidate Phyla Radiation (CPR), discovered mechanisms such as lipid droplet induction, conditional mutualism, and cell-mediated immuno-dampening represent strain-specific features rather than universal ecological laws. Second, a translational gap persists because most mechanistic data—including AI-2 quorum sensing and growth–decline–recovery kinetics—originates from reductionist, static, binary in vitro co-cultures. While essential for dissecting direct molecular interactions, these models fail to capture the complex in vivo macro-environment governed by polymicrobial competition, immune pressures, and nutrient dynamics. Nevertheless, preclinical validation in murine periodontitis models demonstrates that certain protective phenotypes—like the attenuation of alveolar bone loss—are partially preserved within complex biomes. Third, an epidemiological “network bias” arises from the concentration of authorship among a tightly knit network of pioneering research groups, driven by the technical difficulty of co-cultivation pipelines. Independent replication remains scarce, necessitating open-source protocols to validate wild and geographically diverse isolates. Finally, the true translatability of TM7x temporal life-cycle kinetics remains largely hypothetical. In native human oral biofilms, the three-dimensional matrix and interspecies competition may buffer initial physiological shocks, restricting the acute lytic death phase to specific micro-niches populated by unexposed, permissive hosts. Consequently, the established kinetic model should be interpreted as a map of biological potentiality rather than an absolute chronology of natural biofilm progression.

## 6. Conclusions

A systematic synthesis of the evidence to date suggests that *Nanosynbacter lyticus* strain TM7x functions primarily as a regulatory episymbiont within specific human oral microenvironments. Although it retains an initial parasitic phase in naïve hosts, its long-term integration into the microbiome facilitates mutualistic trends that enhance ecological stability and host immune protection. This organism has successfully transitioned from being regarded as “biological dark matter” to serving as a pivotal model for understanding co-evolution within the human microbiome. Ultimately, while its capacity to shield the host from viral predation, induce structurally robust biofilms, and attenuate destructive inflammatory responses positions TM7x as a vital component of the oral homeostatic network, its definitive clinical validity remains potential and contingent upon future validation across broader human clinical contexts.

## 7. Future Perspectives

The multifaceted ecological role of *Nanosymbacter lyticus* TM7x opens critical pathways for future translational research across six strategic domains. First, uncovering bioenergetic horizontal transmission pathways is essential to determine how the ADS and glycolysis maintain infectivity within dynamic salivary environments. Second, evaluating specific Saccharibacteria strains as Next-Generation Probiotics (NGPs) offers novel biotherapies to tame oral pathobiont virulence and restore inflammatory microbial homeostasis. Third, identifying the exact cell-envelope molecular signatures on Actinomyces that dictate host permissiveness will unlock targeted ecological engineering strategies within complex oral biofilms. To overcome current model limitations, fourth, isolation and co-cultivation pipelines must expand to include novel Saccharibacteria phylotypes associated with alternative oral genera (e.g., Streptococcus, Propionibacterium), confirming whether these protective mechanisms are phylum-wide or strain-specific features. Fifth, a methodological migration toward multi-species biofilm models integrating “red complex” pathogens (e.g., Porphyromonas gingivalis) under dynamic microfluidic flow conditions—coupled with human gingival organoids—is crucial to rigorously validate pili-dependent immunomodulation. Finally, bridging the gap from preclinical mechanisms to clinical realities requires transitioning from rodent models toward well-controlled, human longitudinal cohort studies. Determining whether early childhood colonization of the TM7x-Schaalia consortium naturally correlates with long-term resistance to dental caries or periodontitis is a clinical priority before initiating biotherapeutic trials in human populations.

## Figures and Tables

**Table 1:** Comprehensive Summary of Primary Evidence Utilized in the Structured Review.

Citation No.	First Author (Year)	Document/Study Title	Core Variables/Extracted Data Contribution
1	Bor, B. (2019)	Saccharibacteria (TM7) in the Human Oral Microbiome [1].	Baseline epidemiological metrics; phylogenetic classification; 1% core abundance benchmark.
2	Castelle, C.J. (2018)	Major new microbial groups expand diversity and alter our understanding of the tree of life [2].	Candidate Phyla Radiation (CPR)/Patescibacteria superphylum evolutionary context.
3	McLean, J.S. (2016)	Draft Genome Sequence of Actinomyces odontolyticus subsp. actinosynbacter Strain XH001 [3].	Baseline genomic blueprints of the basibiont *Schaalia (Actinomyces) odontolytica* strain XH001.
4	Liu, B. (2012)	Deep sequencing of the oral microbiome reveals signatures of periodontal disease [4].	Core deep-sequencing 16S metadata connecting TM7 operational taxonomic units to active periodontitis.
5	Rylev, M. (2011)	Microbiological and immunological characteristics of young Moroccan patients with aggressive periodontitis [5].	Abundance dynamics of subgingival Candidate Division TM7 in highly aggressive periodontal destruction.
6	Sousa, V. (2017)	Peri-implant and periodontal microbiome diversity in aggressive periodontitis patients: a pilot study [6].	Characterization of total TM7 shifts within active peri-implantitis and aggressive periodontitis cohorts.
7	McLean, J.S. (2020)	Acquisition and Adaptation of Ultra-small Parasitic Reduced Genome Bacteria to Mammalian Hosts [7].	High-resolution genomic scaling (~700 kb) and mapping of core auxotrophies in extracellular parasites.
8	Kindaichi, T. (2016)	Phylogenetic diversity and ecophysiology of candidate phylum Saccharibacteria in activated sludge [8].	Environmental comparative metabolic pathways; evolutionary trajectory of biosynthetic constraints.
9	Hendrickson, E.L. (2022)	Transcriptoma de la cepa epibionte Saccharibacteria Nanosynbacter lyticus TM7x durante el establecimiento de la symbiosis [9].	Comprehensive transcriptomic profiling; kinetic transition states; multi-subunit transporter upregulation.
10	Nahar, N. (2026)	Ultrasmall episymbiont *Nanosynbacter lyticus* employs multiple ATP-generating metabolic pathways during horizontal transmission [10]	Metabolic autonomy; characterization of the Arginine Deiminase System and glycolytic flux during horizontal transfer.
11	Tian, J. (2022)	Acquisition of the arginine deiminase system benefits epiparasitic Saccharibacteria and their host bacteria [11].	Ammonia (NH_3_) and ornithine production mechanics; localized microenvironment pH alkalinization dynamics.
12	Utter, D.R. (2020)	The Saccharibacterium TM7x elicits differential responses across its host range [12].	Host-range mapping; parameters governing permissive versus non-permissive/resistant phenotypes.
13	Zhong, Q. (2024)	Episymbiotic Saccharibacteria TM7x modulates the susceptibility of its host bacteria to phage infection and promotes their coexistence [13].	Epibiotic shield dynamics against lytic phage LC001; surface receptor remodeling (CWP and teichoic acid).
14	Bedree, J.K. (2018)	Quorum Sensing Modulates the Epibiotic-Parasitic Relationship Between Actinomyces odontolyticus and Its Saccharibacteria epibiont.	Autoinducer-2 (AI-2) quorum sensing; deletion phenotypes of *lsrB* and *luxS* loci; biofilm volume expansion.
15	He, X. (2015)	Cultivation of a human-associated TM7 phylotype reveals a reduced genome and epibiotic parasitic lifestyle [14].	Groundbreaking first cultivation protocols for strain TM7x; characterization of the cell-level parasite lifestyle.
16	Shkoporov, A.N. (2022)	Mutualistic interplay between bacteriophages and bacteria in the human gut [15].	Theoretical models for viral predation buffering and community-wide spatial sanctuaries in human microbiomes.
17	Brinig, M.M. (2003)	Prevalence of bacteria of division TM7 in human subgingival plaque and their association with disease [16].	Classic molecular epidemiology mapping subgingival spatial distribution and initial mucosal clinical correlations.
18	Fredricks, D.N. (2005)	Molecular identification of bacteria associated with bacterial vaginosis [17].	Detection and prevalence indices of extra-oral mammalian TM7 sequences in dysbiotic mucosal conditions.
19	Kumar, S. (2008)	Intestinal TM7 bacterial phylogenies in active inflammatory bowel disease [18].	Profiling of Candidate Division TM7 community expansion inside inflamed human gastrointestinal biopsies.
20	Chouhan, D. (2025)	Episymbiotic Saccharibacteria suppresses epithelial immunoactivation through Type IV pili and TLR2 dependent endocytosis [19].	Innate immune response damping; TLR2 clustering; lysosomal trafficking and long-term endocytic survival.
21	Chipashvili, O. (2021)	Episymbiotic Saccharibacteria suppresses gingival inflammation and bone loss in mice through host bacterial modulation [20].	In vivo ligature-induced periodontitis mouse models; quantification of alveolar bone loss; suppression of TNF-α.
22	Camelo-Castillo, A.J. (2015)	Subgingival microbiota in health compared to periodontitis and the influence of smoking [21].	Inter-species micro-niche modeling; relative abundance indices in active tobacco-associated periodontitis.
23	Nowicki, E.M. (2018)	Microbiota and metatranscriptome changes accompanying the onset of gingivitis [22].	Metatranscriptomic shifts capturing the immediate timeline of acute inflammatory development in vivo.
24	Huang, S. (2016)	Microbiota-based signature of gingivitis treatments: a randomized study [23].	Longitudinal clearance kinetics; tracking TM7 therapeutic burden reductions following scaling and root planing.
25	Paster, B.J. (2002)	Bacterial diversity in necrotizing ulcerative periodontitis in HIV-positive subjects [24].	Physical mapping and identification of ultra-deep subgingival TM7 cells adjacent to crevicular epithelium.
26	Eckburg, P.B. (2005)	Diversity of the human intestinal microbial flora [25].	Comparative human baseline distribution data across non-oral healthy mucosal ecosystems.
27	Gao, Z. (2007)	Molecular analysis of human forearm superficial skin bacterial biota [26].	Structural mapping of environmental/mammalian boundary niche diversity of cutaneous candidate divisions.
28	Bor, B. (2018)	Rapid evolution of decreased host susceptibility drives a stable relationship between ultrasmall parasite TM7x [27].	Micro-evolutionary kinetics; multi-generational mutations in host transport proteins; host killing metrics (>50 cells).

**Table 2:** Kinetic Phases and Mechanistic Dynamics of the Epibiotic Interaction Between *N. lyticus* TM7x and *S. odontolytica* XH001.

Phase	Estimated Duration	Host (*S. odontolytica* XH001) Responses	TM7x Activity & Dynamics	References
Initial Encounter	Immediate physical contact	Induction of cellular stress-response genes; subtle morphological alterations.	Upregulation of the Type IV pili system and cell-adhesion proteins.	[9,28]
Death Phase (Lytic Phase)	24–48 h post-infection	Massive physiological shock accompanied by a drastic decline in CFUs; extreme cell elongation and hyphae-like structure formation (growth without division, expanding the surface area available for colonization).	Behavior as a lytic epipatobiont; robust replication via budding, reaching peak consumption of host resources and nutrients.	[9,27]
Recovery	Transition toward microbial equilibrium	Structural resilience and compensation; upregulation of peptidoglycan and rhamnose biosynthetic pathways to remodel the cell envelope and thicken the cell wall.	Downregulation of stress-associated genes; strategic metabolic shift oriented toward persistence and survival.	[9,27]
Stable Symbiosis	Long-term (stationary phase co-culture)	Establishment of a stable biotrophic state, with sustained cell-wall thickening and a reduced but steady growth rate.	Expression of Type IV effector systems; establishment of metabolic homeostasis and stable replication via budding within a heterogeneous cellular community.	[9,10,27]

## Data Availability

The authors confirm that all primary data supporting the findings of this study are derived from peer-reviewed literature and are fully integrated into the article. Specifically, the comprehensive list of analyzed sources, extraction strategies, and synthesized variables is detailed in Section 2 and Table 1 and Table 2. No new raw biological or clinical datasets were generated during this review. The specific text prompts utilized for the generative design of the manuscript’s illustrations are proprietary and are not publicly available, thereby protecting original creative workflows. Further inquiries regarding the literature selection criteria can be directed to the corresponding author.

## Abbreviations

The following abbreviations are used in this manuscript:

Abbreviation	Full Definition
ADS	Arginine Deiminase System
AI-2	Autoinducer-2
ATP	Adenosine Triphosphate
CFU	Colony Forming Units
COPE	Committee on Publication Ethics
CPR	Candidate Phyla Radiation
CWP	Cell Wall Polysaccharides
IBD	Inflammatory Bowel Disease
Kb	Kilobase (or kilobase pairs)
LsrB	LuxS-regulated periplasmic binding protein
LuxS	S-ribosylhomocysteine lyase (enzyme responsible for AI-2 synthesis)
NGPs	Next-Generation Probiotics
NH_3_	Ammonium
Nm	Nanometers
PRISMA	Preferred Reporting Items for Systematic Reviews and Meta-Analyses
rRNA	Ribosomal Ribonucleic Acid
SecA	Secretory protein A (translocase subunit)
TLR2	Toll-Like Receptor 2
TM7	Candidate Division TM7 (now Saccharibacteria)
TNF-α	Tumor Necrosis Factor-alpha
UgpC	Sn-glycerol-3-phosphate transport system permease protein
